# Association between lifestyle habits and glaucoma incidence: a retrospective cohort study

**DOI:** 10.1038/s41433-023-02535-7

**Published:** 2023-04-19

**Authors:** Asahi Fujita, Yohei Hashimoto, Hiroki Matsui, Hideo Yasunaga, Makoto Aihara

**Affiliations:** 1 Department of Ophthalmology, Graduate School of Medicine, The University of Tokyo, Tokyo, Japan; 2 Department of Clinical Epidemiology and Health Economics, School of Public Health, The University of Tokyo, Tokyo, Japan

**Keywords:** Risk factors, Vision disorders

## Abstract

**Background/Objectives:**

Although lifestyle habits may represent modifiable risk factors of glaucoma, the association between lifestyle factors and glaucoma is not well understood. The aim of this study was to investigate the association between lifestyle habits and the development of glaucoma.

**Subjects/Methods:**

Participants who underwent health check-ups from 2005 to 2020 using a large-scale administrative claims database in Japan were included in the study. Cox regression analyses were performed where glaucoma development was regressed on the lifestyle (body mass index, current smoking, frequency and amount of alcohol consumption, eating habits, exercise habits and quality of sleep), age, sex, hypertension, diabetes mellitus and dyslipidaemia.

**Results:**

Among the 3,110,743 eligible individuals, 39,975 developed glaucoma during the mean follow-up of 2058 days. Factors associated with increased risk of glaucoma were overweight/obese (vs. moderate weight: hazard ratio, 1.04 [95% confidence interval, 1.02–1.07]), alcohol consumption of 2.5–4.9 units/day, 5–7.4 units/day, and ≥7.5 units/day (vs. <2.5 units/day: 1.05 [1.02–1.08], 1.05 [1.01–1.08] and 1.06 [1.01–1.12], respectively), skipping breakfast (1.14 [1.10–1.17]), late dinner (1.05 [1.03-1.08]) and daily walking of 1 h (1.14 [1.11–1.16]). Factors associated with decreased risk of glaucoma were daily alcohol consumption (vs. rarely: 0.94 [0.91–0.97]) and regular exercise (0.92 [0.90–0.95]).

**Conclusions:**

Moderate body mass index, having breakfast, avoiding late dinner, limiting alcohol intake to <2.5 units/day, and regular exercise were associated with a reduced risk of developing glaucoma in the Japanese population. These findings may be useful for promoting glaucoma prophylaxis.

## Introduction

Glaucoma is the most common cause of irreversible blindness worldwide [1]. Widely accepted risk factors for glaucoma include old age, ethnic background, a family history of glaucoma, and high myopia [1, 2]. However, modifying these known risk factors is challenging.

Lifestyle habits are modifiable when individuals recognize the risk of diseases and work toward their improvement. Numerous studies have investigated the association between lifestyle habits and glaucoma; however, they demonstrated conflicting results concerning body mass index (BMI), cigarette smoking and alcohol consumption [3–11]. Moderate exercise is the only lifestyle factor that has been consistently associated with a reduced risk of glaucoma [12, 13]. In addition, no previous studies have examined the association of eating habits and quality of sleep with the incidence of glaucoma.

This study aimed to investigate the association between lifestyle habits (including smoking, alcohol consumption, BMI, eating habits, exercise habits and the quality of sleep) and the development of glaucoma, using a large database of health-check-ups and administrative claims data in Japan.

## Subjects and methods

### Data source

We used the JMDC Claims Database (JMDC Inc., Tokyo, Japan) between 2005 and 2020. This database consists of annual health check-ups data and administrative claims data of hospital admission and clinic visits, which are collected from company employees and their families aged between 0 and 74. Employers are mandated to perform regular health check-ups for their employees in Japan. Administrative claims data include the data on the diagnoses based on the International Classification of Diseases Tenth Revision (ICD-10) and drugs dispensed. Annual health check-up data include the data on BMI, blood pressure, fasting glucose level and self-reported health questionnaires on the smoking status (“current smoker” or “non-current smoker [non-smoker and ex-smoker]”), frequency of alcohol consumption (“rarely,” “sometimes” or “daily”), amount of Japanese sake equivalent alcohol consumption (“<180 ml/day,” “180–359 ml/day,” “360–539 ml/day” and “≥540 ml/day”), eating habits, exercise habits and quality of sleep. Self-reported health questionnaires have frequently been used to examine these exposure variables in previous clinical epidemiological studies [14]. The details of the questions concerning the eating habits, exercise habits and the quality of sleep are as follows: (1) “Do you skip breakfast three or more times a week? (skipping breakfast),” (2) “Do you have dinner within 2 h of bedtime three or more times a week? (late dinner),” (3) “Do you walk at least 1 h a day? (daily walking),” (4) “Have you been in the habit of regular exercise of over 30 min about twice a week for more than a year? (regular exercise)” (5) “Are you getting enough rest with sleep? (good quality of sleep).”

### Study design and patient selection

**Fig. 1:**
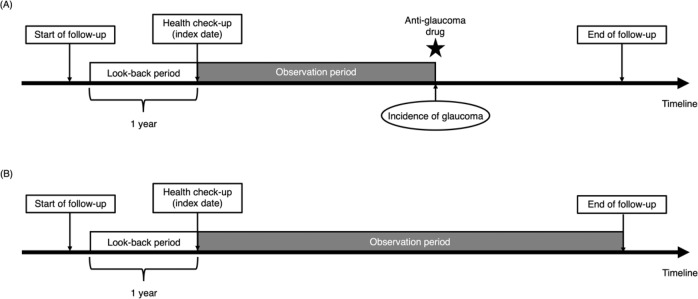
Study design. **A** Individuals who developed glaucoma, **B** individuals who did not develop glaucoma.

We included individuals who underwent annual health check-ups with look-back periods of 1 year. A look-back period is a disease-free period leading up to the start of the observation. We excluded individuals aged under 20 years and those with missing data on any of the following variables: hypertension, diabetes mellitus, dyslipidaemia, BMI, smoking status, drinking status, eating habits, exercise habits and quality of sleep. We also excluded those with pre-existing glaucoma, defined as those who had diagnostic records of glaucoma (ICD-10 codes, H40.1-H40.9, H42.0, H42.8 and Q15.0) within a 1-year look-back period. We employed this definition of pre-existing glaucoma because this definition had higher sensitivity than those using prescription codes or procedure codes [15]. The index date was defined as the date of the first health check-up following the 1-year look-back period. For individuals who developed glaucoma during the follow-up period, the observation ended on the day of glaucoma development (Fig. 1A). For individuals who did not develop glaucoma during the follow-up period, the observation was censored on the last day of follow-up (Fig. 1B).

### Outcome, exposures, and covariates

The outcome was the incidence of glaucoma, defined as having at least one dispensing record of antiglaucoma eyedrops (Supplementary Table). We employed the definition using dispensing records of antiglaucoma eyedrops for the outcome because this definition had a higher specificity and positive predictive value than that using diagnostic codes [15]. We defined exposure as follows: BMI was categorized into three groups based on the World Health Organization recommended BMI cut-off points for the Japanese: “underweight (<18.5 kg/m^2^),” “moderate (≥18.5 kg/m^2^ and <25 kg/m^2^)” “overweight or obese (≥25 kg/m^2^)” [16]. Smoking status (“current smoker” or “non-current smoker [non-smoker and ex-smoker]”), breakfast (“skipping” or “not skipping”), dinner (“late” or “not late”), walking (“daily” or “not daily”), exercise (“regular” or “not regular”), and quality of sleep (“good” or “not good”) were defined based on the self-reported health questionnaire data. Frequency of alcohol consumption was categorized as “rarely,” “sometimes” or “daily.” The amount of alcohol consumption was categorized based on Japanese sake-equivalent quantity as “<180 ml/day,” “180–359 ml/day,” “360–539 ml/day” and “≥540 ml/day.” Japanese sake usually has an alcohol concentration of 15%, roughly equivalent to red wine. A 180 ml bottle of Japanese sake contains 2.5 alcohol units. One alcohol unit (the amount of alcohol an average adult can process in an hour) equals 10 ml or 8 g of pure alcohol. The covariates were age, sex, and history of hypertension (having a systolic blood pressure of ≥140 mmHg, a diastolic blood pressure of ≥90 mmHg or an antihypertensive prescription), diabetes mellitus (having a fasting glucose level ≥126 mg/dl or an antidiabetic prescription) and dyslipidaemia (having a low-density lipoprotein cholesterol level ≥140 mg/dl, a high-density lipoprotein cholesterol level <40 mg/dl, triglycerides ≥150 mg/dl or a lipid-lowering prescription).

### Statistical analysis

First, we estimated the crude hazard ratios (HRs) and their 95% confidence intervals (CIs) for glaucoma development using univariable Cox regression, with the regression of glaucoma development on each exposure. Second, we estimated the adjusted HRs and their 95% CIs using multivariable Cox regression in which glaucoma development was regressed on all the exposures, age, sex, hypertension, diabetes mellitus and dyslipidaemia. We calculated the variance inflation factors to test the multicollinearity of the multivariable Cox model. We considered the variance inflation factors greater than 10 as having multicollinearity. Because sex may affect the association between lifestyle and glaucoma, we included the interaction terms between sex and lifestyle into the multivariable Cox model. Furthermore, we performed multivariable Cox regression stratified by sex. Statistical analyses were performed using R software version 3.6.1 (R Foundation for Statistical Computing, Vienna, Austria). A significance level of 5% was used for all the analyses.

## Results

### Participant selection and characteristics

**Fig. 2:**
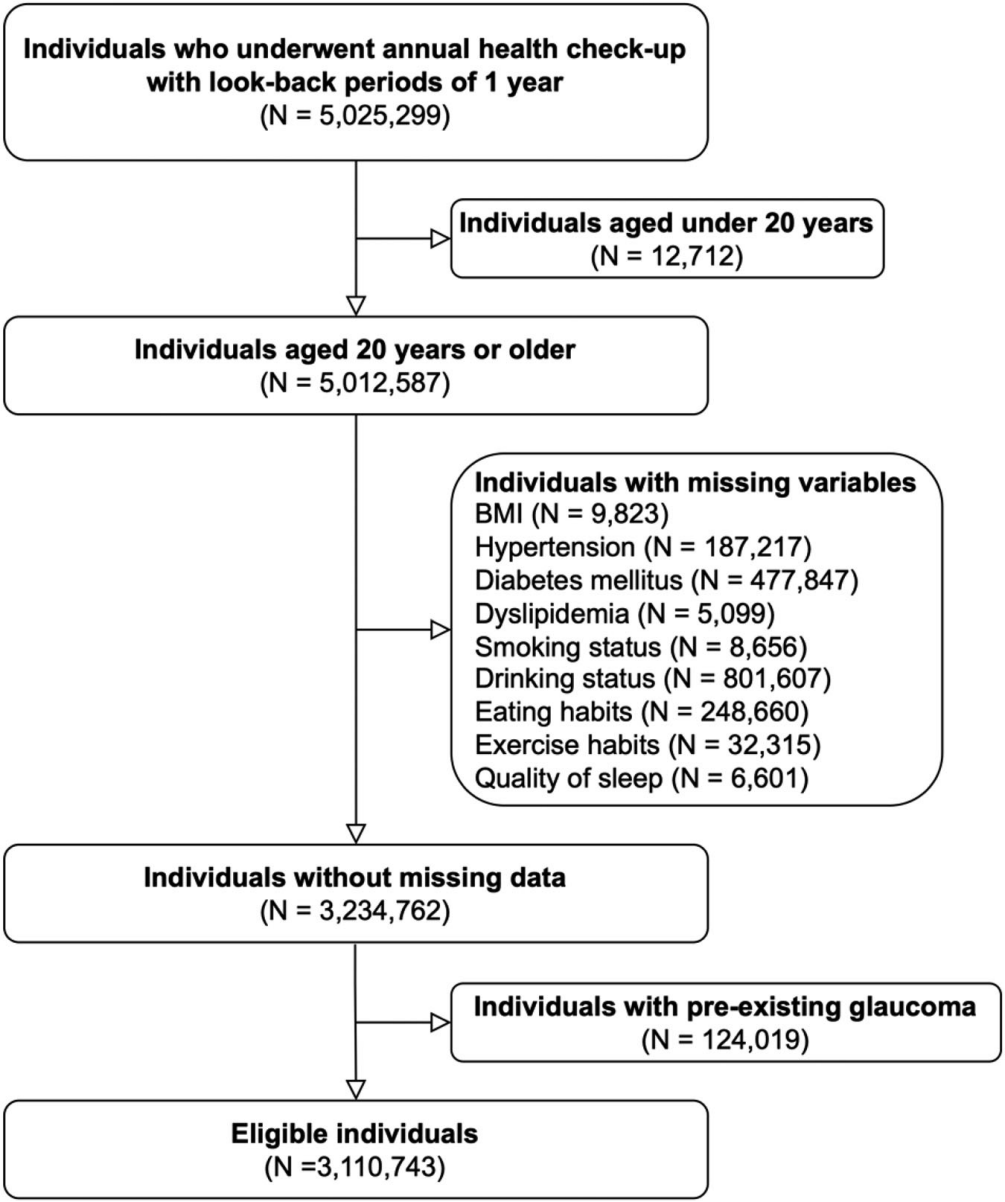
Participant selection. We identified 3,110,743 eligible individuals.

A flowchart of participant selection for the main analysis is shown in Fig. 2. We identified 3,110,743 eligible individuals among whom 39,975 developed glaucoma during the observation period (Table 1). The mean age at the start of the observation was 44.4 years old, and males accounted for 61.7%. The mean follow-up period was 2058 days.

**Table 1:** Participant characteristics.

Number of participants	31,10,743
Glaucoma development (%)	39,975 (1.3)
Age (years, mean [SD])	44.4 (11.1)
Age category (%)	
20–29	343,958 (11.1)
30–39	656,199 (21.1)
40–49	1,070,763 (34.4)
50–59	739,108 (23.8)
60–69	278,304 (8.9)
70–75	22,411 (0.7)
Male (%)	1,917,939 (61.7)
Follow-up period (days, mean [SD])	2057.9 (1077.9)
Hypertension (%)	624,201 (20.1)
Diabetes mellitus (%)	153,464 (4.9)
Dyslipidemia (%)	1,255,653 (40.4)
BMI category (%)	
<18.5 kg/m^2^, Underweight	246,996 (7.9)
≥18.5 kg/m^2^ and <25 kg/m^2^, Moderate	2,067,085 (66.4)
≥25 kg/m^2^, Overweight or obese	796,662 (25.6)
Current smoking (%)	825,809 (26.5)
Frequency of alcohol consumption (%)	
Rarely	887,205 (28.5)
Sometimes	1,390,144 (44.7)
Daily	833,394 (26.8)
Amount of sake equivalent alcohol consumption (%)	
<180 ml (2.5 units)/day	1,672,946 (53.8)
180–359 ml (2.5–4.9 units)/day	881,743 (28.3)
360–539 ml (5.0–7.4 units)/day	399,232 (12.8)
≥540 ml (7.5 units)/day	156,822 (5.0)
Skipping breakfast (%)	784,708 (25.2)
Late dinner (%)	1,157,806 (37.2)
Daily walking for 1 h (%)	1,248,417 (40.1)
Thirty minutes of exercise twice a week (%)	644,929 (20.7)
Good quality of sleep (%)	1,907,983 (61.3)

*SD* standard deviation, *BMI* body mass index.

### Crude HRs for glaucoma development

**Fig. 3:**
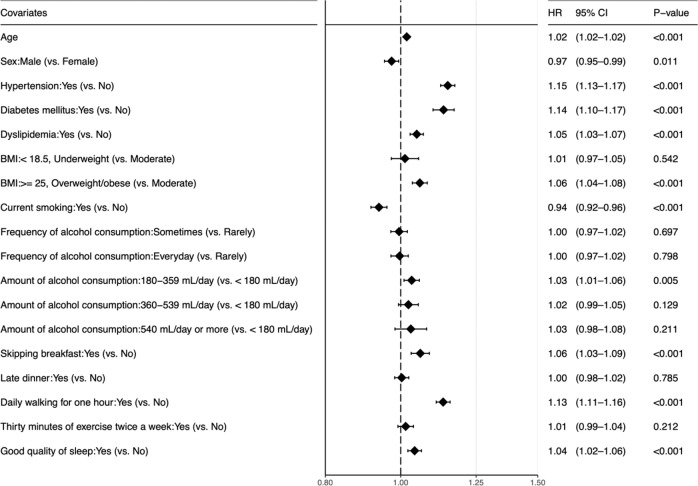
Crude hazard ratios of each covariate for glaucoma development. The amount of alcohol is shown in servings of Japanese sake, similar to red wine in alcohol content. A 180 ml bottle of Japanese sake contains 2.5 alcohol units. HR hazard ratio, CI confidence interval, BMI body mass index.

Figure 3 shows the results of univariate Cox regression. Overweight or obese (vs. Moderate: HR, 1.06 [95% CI, 1.04–1.08]), 180–359 ml/day of alcohol consumption (vs. <180 ml/day; 1.03 [1.01–1.06]), skipping breakfast (1.06 [1.03–1.09]), daily walking for 1 h (1.13 [1.11–1.16]) and good quality of sleep (1.04 [1.02–1.06]) were associated with increased risk of glaucoma. Current smoking (0.94 [0.92–0.96]) was inversely associated with glaucoma development compared to non-current smoking.

### Adjusted HRs for glaucoma development

**Fig. 4:**
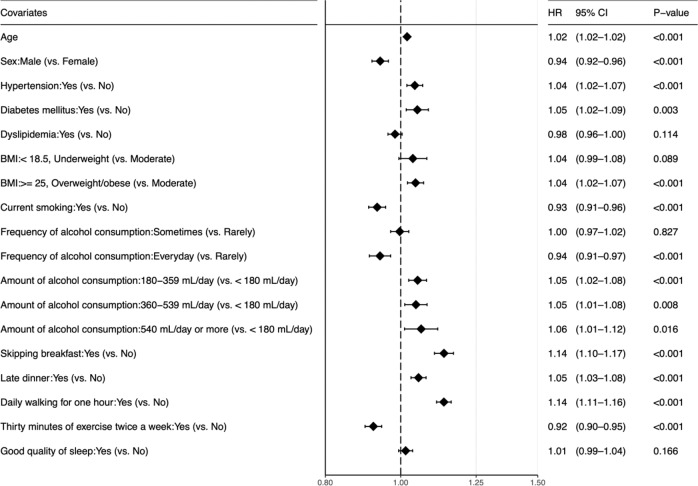
Adjusted hazard ratios of each covariate for glaucoma development. The amount of alcohol is shown in servings of Japanese sake, similar to red wine in alcohol content. A 180 ml bottle of Japanese sake contains 2.5 alcohol units. HR hazard ratio, CI confidence interval, BMI body mass index.

The variance inflation factors of the regression model were less than 2 for all the covariates, indicating that multicollinearity did not exist. Therefore, we included all the variables of interest in the multivariable Cox regression model. The results are presented in Fig. 4. Increased risk of glaucoma was associated with overweight/obese (vs. Moderate: HR, 1.04 [95% CI, 1.02–1.07]), 180–359 ml/day, 360–539 ml/day and ≥540 ml/day of alcohol consumption (vs. <180 ml/day: 1.05 [1.02–1.08], 1.05 [1.01–1.08] and 1.06 [1.01–1.12], respectively), skipping breakfast (1.14 [1.10–1.17]), late dinner (1.05 [1.03–1.08]) and daily walking for 1 h (1.14 [1.11–1.16]). Current smoking (vs. non-current smoking, 0.93 [0.91–0.96]), daily alcohol consumption (vs. Rarely, 0.94 [0.91–0.97]) and regular exercise (0.92 [0.90–0.95]) were inversely associated with glaucoma incidence.

### Adjusted HRs for glaucoma development stratified by sex

The results of the multivariable Cox regression stratified by sex are shown in Supplementary Fig. Overweight/obese was significantly associated with an increased risk of glaucoma in both males and females (vs. Moderate: HR, 1.03 [95% CI, 1.00–1.06] and 1.05 [1.00–1.11], respectively). Underweight was significantly associated with glaucoma development in females; however, the association was not significant in males (vs. Moderate: 1.05 [1.00–1.11] and 1.02 [0.95–1.09], respectively). Regarding the frequency of alcohol consumption, both occasional and daily consumptions were associated with reduced risks of glaucoma in males (vs. Rarely: 0.95 [0.92–0.99] and 0.91 [0.87–0.94], respectively), whereas neither of them had significant associations with glaucoma in females (1.03 [0.99–1.07] and 0.97 [0.92–1.03], respectively). Regarding the amount of alcohol consumption, 180–359 ml/day, 360–539 ml/day and ≥540 ml/day were significantly associated with increased risk of glaucoma in females (vs. <180 ml/day; 1.14 [1.08–1.20], 1.12 [1.03–1.22] and 1.21 [1.04–1.40], respectively). They tended to have similar associations in males; however, the associations were not statistically significant. Late dinner was associated with an increased risk of glaucoma in both males and females, and the association was stronger in females compared to males (*p* for interaction, 0.001). The patterns of the associations for skipping breakfast, daily walking, regular exercise and quality of sleep were similar between males and females.

## Discussion

We examined the association between overall lifestyle (including eating habits and sleep quality) and glaucoma using large-scale longitudinal data. We revealed that overweight/obese, ≥2.5 units/day of alcohol consumption compared to <2.5 units/day, skipping breakfast, late dinner and daily walking were associated with increased incidence of glaucoma after adjusting for baseline characteristics. In contrast, daily alcohol consumption and regular exercise were associated with reduced incidence of glaucoma.

### Overweight/obese

In the current study, overweight/obesity significantly increased the risk of glaucoma both in males and females. Some previous studies reported that obesity reduced the risk of glaucoma [3–5]; however, another study reported that it increased the risk of glaucoma [6]. These conflicting results between studies were attributed to different sociodemographic characteristics of the study population, different definitions of obesity and multiple metabolisms, such as insulin resistance involved in the obesity spectrum [6]. Our results of overweight/obesity were similar to those in Korea [6], and different from those in the Netherlands [3], in the U.S. [4] and those of African Americans [5]. Therefore, we speculate that staying non-obese would work positively to prevent glaucoma for Asian individuals. We presume that the inconsistent association between obesity and glaucoma among the studies were partly owing to the different subtypes of glaucoma prevalent in each population. Unlike in European countries and the U.S., normal tension glaucoma (NTG) is the most prevalent subtype of glaucoma in Asia [17]. NTG is different from other subtypes of glaucoma in that it is not accompanied by increased intraocular pressure, a major cause of glaucoma. Rather, possible causes of NTG include a higher sensitivity to normal pressure, vascular dysregulation, an abnormally high translaminar pressure gradient and a neurodegenerative process due to impaired cerebrospinal fluid dynamics in the optic nerve sheath compartment. The pathogenesis of NTG could be related to obesity; however, further research on the mechanisms of NTG is warranted.

### Underweight

Being underweight was associated with a significantly increased risk of glaucoma only in females. Underweight females have less adipose tissue, which is the major place of estrogen production especially for postmenopausal women [18]. Recently, activation of G protein coupled estrogen receptors exhibited neuroprotective effects against retinal ganglion cell degeneration [19]. Thus, we speculate that insufficient plasma estrogen in underweight females could result in retinal ganglion cell degeneration, which is known to play a key role in the pathogenesis of glaucoma.

### Current smoking

In our study, current smoking was associated with a reduced risk of glaucoma compared to non-current smoking. However, in the database used in our study, non-current smokers included both non-smokers and ex-smokers. Moreover, the database lacked data on the smoking volume and duration. Thus, we are unable to draw any conclusion regarding the association between smoking and glaucoma based on our results. Previous studies demonstrated inconsistent results concerning the association between smoking and glaucoma [3, 7, 8]. A previous study reported that the risk of glaucoma was higher in ex-smokers compared to non-smokers and current smokers [9]. The authors speculated that ex-smokers might have consumed a large number of cigarettes resulting in serious medical conditions related to smoking, and they may have been motivated to quit smoking. Among smokers, a greater number of cigarette packs consumed per day was associated with higher odds of glaucoma [9].

### Alcohol consumption

Daily alcohol consumption was associated with a reduced risk of glaucoma. Alcohol is known to have a temporal ocular hypotensive effect, with a peak within 1–3 h following ingestion [10, 11]. Since most people consume alcohol in the evening in Japan, these transient hypotensive effects usually act at night. Cumulative evidence has demonstrated that intraocular pressure peaks at night [20, 21]. Therefore, daily intake of alcohol in the evening may help reduce the peak of intraocular pressure, possibly resulting in prevention of the development of glaucoma. On the other hand, daily volume of alcohol consumption ≥180 ml (2.5 units)/day was associated with increased risk of glaucoma compared to <180 ml/day. Our results were similar to a recent analysis of the UK Biobank that reported that regular drinkers with a greater quantity of alcohol intake demonstrated a higher prevalence of glaucoma in a dose-dependent manner [22]. Chronic excessive alcohol use is known to cause neuropathy through oxidative stress resulting in free radical damage on nerves, nutritional deficiencies, and direct toxic effects, and alcoholic neuropathy is more prevalent in heavy drinkers [23]. Excessive intake of alcohol may damage the optic nerves by mechanisms similar to alcoholic neuropathy. Our results suggested that females tended to be more susceptible to glaucoma than males in terms of alcohol consumption. A previous study showed that females were more susceptible to long-term negative effects of alcohol on health, and developed alcohol-related medical conditions after shorter duration and at lower levels of alcohol consumption compared to males [24]. A meta-analysis revealed that the relative risk of all-cause mortality significantly increased in females who consumed 2.0–2.9 standard drinks per day compared to abstainers, whereas it reached significance only when 4 standard drinks per day were consumed in males [25]. Sex-related biological factors, including differences in alcohol pharmacokinetics and the levels of sex hormones, are considered to contribute to this difference in susceptibility to various medical condition [24].

### Eating habits

Both skipping breakfast and late dinner were significantly associated with an increased risk of glaucoma. Accumulating evidence suggested that skipping breakfast increased the risk of cardiovascular disease [26]. A previous study reported that habitual skipping of breakfast resulted in blood pressure elevation in the morning and altered lipid profiles, thereby increasing the risk of cardiovascular diseases [27]. Having late dinner was also associated with an increased risk of cardiovascular disease [28]. In addition, late dinner reportedly resulted in high blood glucose levels in the following morning [29]. Skipping breakfast and late dinner may predispose to glaucoma through elevated blood pressure and blood glucose levels, since both of them are risk factors for glaucoma [30, 31]. Late dinner was more strongly associated with an increased risk of glaucoma in females compared to males. In a previous study, circadian misalignment increased the risk of obesity in females, but not in males [32]. Thus, females may be prone to medical conditions in disrupted rhythm of life owing to sex-dependent factors, such as sex hormone malfunction.

### Walking and exercise

Exercising for more than 30 min twice a week was significantly associated with lower risk of glaucoma, whereas walking for more than 1 h every day was significantly associated with a higher risk of glaucoma. In a previous study, both high and low intensity of exercise were associated with greater glaucoma prevalence compared to moderate intensity of exercise [12]. They also reported that daily vigorous exercise was associated with increased prevalence of glaucoma compared to vigorous exercise performed 3 days per week. Another study reported that moderate amounts of vigorous activity decreased the risk of glaucoma compared to no vigorous activity [13]. Thus, exercise would have both positive and negative effects on the development of glaucoma depending on the intensity and frequency. Excessive exercise has been associated with the accumulation of free radicals that cause structural damage and inflammatory reactions, and this oxidative stress could trigger glaucoma [33]. On the contrary, neuroprotective effects of exercise have also been reported. In animal models, exercise has been shown to increase the expression of brain-derived neurotrophic factors, enhance mitochondrial function, and reduce oxidative stress in the retina, which could be effective in preventing retinal ganglion cell death [34]. Thus, moderate intensity exercise, and not excessive exercise, could be considered ideal. Our results suggest that 1 h of daily walking may be excessive, and 30 min of exercise twice a week may be considered moderate.

### Quality of sleep

Good quality of sleep was not associated with the incidence of glaucoma. Previous studies on the association between sleep and glaucoma mainly focused on sleep apnea, which remains controversial [35, 36]. Many factors are involved in the pathogenesis of sleep apnea, such as hypoxia, obesity and hormonal imbalance, and each factor is intricately related to glaucoma [35]. Our study revealed that quality of sleep itself was not associated with the development of glaucoma.

Our study has several limitations. First, we included individuals who were aged between 20 and 75 years and reside in Japan. Second, many of the parameters obtained in the annual health check-ups were self-reported, which may be subject to recall bias. Since it is realistically difficult to collect data on the lifestyle habits from millions of individuals, we used these self-reported variables similar to previous clinical epidemiological studies. Third, the cohort included a higher proportion of males than females. We included interaction terms between sex and lifestyle, and we also performed the analyses stratified by sex. Fourth, we did not differentiate between the subtypes of glaucoma. In a previous study using Japanese claims data, the validity of the diagnostic codes to identify specific subtypes of glaucoma (such as primary open angle glaucoma) was low, although the diagnostic validity for glaucoma was high when not specifying the subtype [15]. Fifth, data on the details of food intake and the amount and past history of smoking were not available. Sixth, we investigated the associations between lifestyle habits and glaucoma; however, the causal relationship remains unknown.

In conclusion, moderate BMI, having breakfast, avoiding late dinner, limiting alcohol intake to <2.5 units/day and 30 min of exercise at least twice a day were associated with a reduced risk of developing glaucoma. These findings could be utilized for promoting the prophylaxis of glaucoma.

## Summary

### What was known before

- Glaucoma is the most common cause of irreversible blindness, and it creates a societal burden through the cost of treatment, post-blindness care, and lost productivity.
- Among various lifestyle habits (that can be modified to help individuals manage or decrease their risk), moderate exercise is the only lifestyle factor that has been consistently associated with a reduced risk of glaucoma.

### What this study adds

- Factors associated with increased risk of glaucoma were being overweight/obese (in addition to being underweight in females), ≥2.5 units/day of alcohol, and skipping breakfast and late dinner.
- In contrast, daily alcohol consumption and regular exercise were associated with decreased risk of glaucoma.
- Our findings may be useful for promoting glaucoma prophylaxis and have the potential to reduce the social burden associated with glaucoma.

### Supplementary information

Supplementary Table and Figure

## Data Availability

We used de-identified, individual-level data obtained from the JMDC Claims Database (Tokyo, Japan). The address of their HP is https://www.jmdc.co.jp/en/index. Data are not publicly available.
