# Supplementary material for: Association between lifestyle habits and glaucoma incidence: a retrospective cohort study
Source: Eye (Lond). 2023 Apr 19;37(16):3470–6. doi: 10.1038/s41433-023-02535-7 (PMC10630484; doi:10.1038/s41433-023-02535-7)
Supplement: Supplementary file 1 — Supplementary Table and Figure [file 41433_2023_2535_MOESM1_ESM.docx]

Supplementary Table: List of antiglaucoma eyedrops included in this study

Supplementary Figure: Adjusted hazard ratios of each covariate for glaucoma development stratified by sex

**Supplementary Table. List of antiglaucoma eyedrops included in this study**

| Drug categories | Generic names |
| --- | --- |
| FP agonist | Latanoprost |
|  | Bimatoprost |
|  | Travoprost |
|  | Tafluprost |
|  | Isopropyl Unoprostone |
| EP2 agonist | Omidenepag Isopropyl |
| Non-selective BB | Timolol Maleate |
|  | Carteolol Hydrochloride |
| Selective BB | Betaxolol Hydrochloride |
| CAI | Dorzolamide Hydrochloride |
|  | Brinzolamide |
| ROCK inhibitor | Ripasudil Hydrochloride Hydrate |
| Alpha-2 adrenergic agonist | Brimonidine Tartrate |
| Adrenergic agents | Dipivefrin Hydrochloride |
| Cholinergic agents | Pilocarpine Hydrochloride |
| Cholinesterase inhibitor | Distigmine Bromide |
| Alpha-1 beta adrenergic antagonist | Nipradilol |
|  | Levobunolol Hydrochloride |
| Alpha-1 blocker | Bunazosin Hydrochloride |
| FP agonist/BB | Latanoprost + Timolol Maleate |
|  | Travoprost + Timolol Maleate |
|  | Tafluprost + Timolol Maleate |
|  | Latanoprost + Carteolol Hydrochloride |
| CAI/BB | Dorzolamide Hydrochloride + Timolol Maleate |
|  | Brinzoramide + Timolol Maleate |
| Alpha-2 adrenergic agonist/BB | Brimonidine Tartrate + Timolol Maleate |
| Alpha-2 adrenergic agonist/CAI | Brimonidine Tartrate + Brinzolamide |

FP agonist, prostanoid FP receptor agonist; EP2 agonist, prostanoid EP2 receptor agonist; BB, beta-blocker; CAI, carbonic anhydrase inhibitor; ROCK, rho-associated protein kinase

**Supplementary Figure. Adjusted hazard ratios of each covariate for glaucoma development stratified by sex**

**
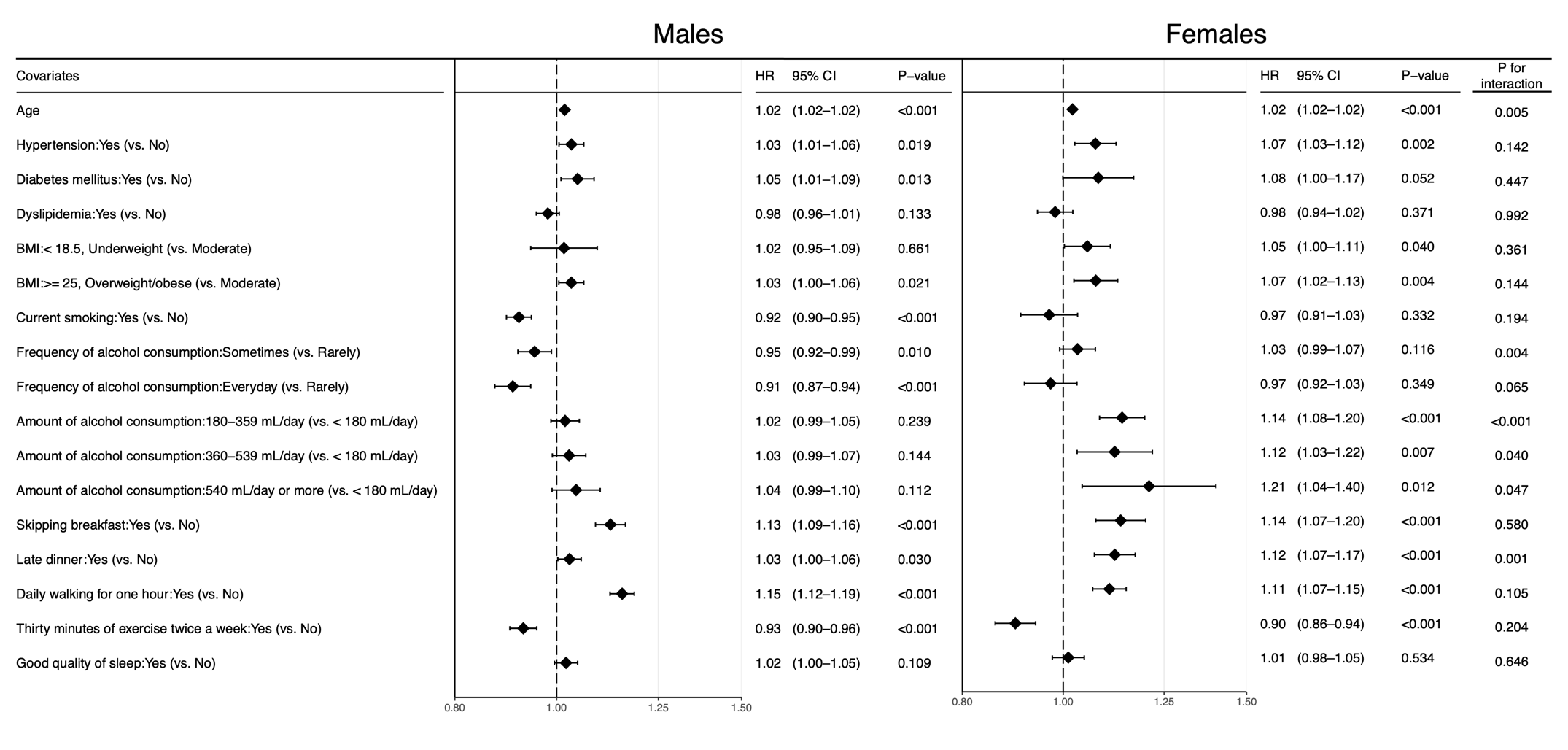
**

The amount of alcohol is shown in Japanese sake equivalent, whose alcohol concentration is almost equal to red wine. A 180 mL bottle of Japanese sake contains 2.5 alcohol units.

HR, hazard ratio; CI, confidence interval; BMI, body mass index
